# Chromium and Ruthenium-Doped Zinc Oxide Thin Films for Propane Sensing Applications

**DOI:** 10.3390/s130303432

**Published:** 2013-03-12

**Authors:** Heberto Gómez-Pozos, José Luis González-Vidal, Gonzalo Alberto Torres, Jorge Rodríguez-Baez, Arturo Maldonado, María de la Luz Olvera, Dwight Roberto Acosta, Maximino Avendaño-Alejo, Luis Castañeda

**Affiliations:** 1 Área académica de Computación, ICBI, Universidad Autónoma del Estado de Hidalgo, Mineral de la Reforma, Hidalgo, Apartaddo Postal 42000, Mexico; E-Mails: gpozos@uaeh.edu.mx (H.G.-P.); jlvidal@uaeh.edu.mx (J.L.G.-V.); torres@uaeh.edu.mx (G.A.T.); jrbaez@prodigy.net.mx (J.R.-B.); 2 Departamento de Ingeniería Eléctrica-SEES, Centro de Investigación y de Estudios Avanzados del Instituto Politécnico Nacional, CINVESTAV-IPN, Apartado Postal 14740, Mexico D.F. 07000, Mexico; E-Mails: amaldo@cinvestav.mx (A.M.); molvera@cinvestav.mx (M.L.O.); 3 Instituto de Física, Universidad Nacional Autónoma de México, Apartado Postal 20-364, México, D.F. 01000, Mexico; E-Mail: dacosta@fisica.unam.mx; 4 Centro de Ciencias Aplicadas y Desarrollo Tecnológico, Universidad Nacional Autónoma de México, Apartado Postal 70-186, 04510, D.F. 07340, Mexico; E-Mail: maximino.avendano@ccadet.unam.mx; 5 Escuela Superior de Ingeniería Mecánica y Eléctrica Unidad Ticomán, Instituto Politécnico Nacional, Mexico, D.F. 07340, Mexico

**Keywords:** zinc oxide, thin solid films, sol-gel, 07.07.Df, 73.61.–r, 52.77.Fv, 81.15.Rs

## Abstract

Chromium and ruthenium-doped zinc oxide (ZnO:Cr) and (ZnO:Ru) thin solid films were deposited on soda-lime glass substrates by the sol-gel dip-coating method. A 0.6 M solution of zinc acetate dihydrate dissolved in 2-methoxyethanol and monoethanolamine was used as basic solution. Chromium (III) acetylacetonate and Ruthenium (III) trichloride were used as doping sources. The Ru incorporation and its distribution profile into the films were proved by the SIMS technique. The morphology and structure of the films were studied by SEM microscopy and X-ray diffraction measurements, respectively. The SEM images show porous surfaces covered by small grains with different grain size, depending on the doping element, and the immersions number into the doping solutions. The sensing properties of ZnO:Cr and ZnO:Ru films in a propane (C_3_H_8_) atmosphere, as a function of the immersions number in the doping solution, have been studied in the present work. The highest sensitivity values were obtained for films doped from five immersions, 5.8 and 900, for ZnO:Cr and ZnO:Ru films, respectively. In order to evidence the catalytic effect of the chromium (Cr) and ruthenium (Ru), the sensing characteristics of undoped ZnO films are reported as well.

## 1. Introduction

Zinc oxide is one of the most important multifunctional semiconductor oxides because of its physical properties, such as resistivity control over the range 10^−3^–10^5^ Ωcm, high transparency in the visible range, chemical and thermal stability at room temperature, a direct wide bandgap, around 3.37 eV, and a large exciton binding energy of 60 meV [1]. These characteristics make ZnO thin films very attractive for different applications, such as, solar cell transparent contacts [2], surface acoustic wave systems [3], liquid crystal displays [4], gas sensors [5,6], and other optoelectronic devices [7].

Nowadays, all kind of gas sensors is widely demanded for a wide variety of domestic and industrial applications, such as, exhaust gas sensing in the automotive industry and flammable and toxic gases, or for monitoring furnace installations. In the last decades, metal-oxide semiconductors have been extensively applied for detecting different polluting gases. Until now, ZnO has been one of the metal-oxide semiconductors most used for gas sensing applications [8,9], due to their low cost of production, high sensitivity, low toxicity level, and low power consumption. The sensing properties of undoped and doped ZnO thin films have been reported for different gases, carbon monoxide (CO) [10], methane (CH_4_) [11], acetylene (C_2_H_2_) [12], and nitrogen dioxide (NO_2_) [13], among others. Although undoped semiconductor oxides are catalytically active, a dopant element (catalyst) is often added to improve their sentitivity and selectivity. In this respect, different elements have been tested as catalysts in ZnO films, and it has been widely documented that an adequate selection of the catalyst, depending on the detecting gases, leads to an improvement of the sensing properties [14–20]. ZnO samples can be processed by different deposition techniques, such as, thermal evaporation [21], sputtering [22], chemical vapor deposition [23], chemical spray [24], and sol- gel technique [25].

The main goal of this research is to present some results about explorative investigations on ZnO thin films deposited by the sol-gel dip-coating technique, doped, separately, with ruthenium (ZnO:Ru) and chromium (ZnO:Cr), in order to test their sensing properties in a controlled atmosphere of gas propane (C_3_H_8_) As, most of the time, the selection of the semiconductor oxide characteristics, for best performance, in a specific application has usually an empirical character, therefore, it is very important to know the parameters of ZnO samples, which can be used in sensor applications. In this respect, the sensitivity variation as a function of the film thickness, controlled by the immersions number, the propane concentration, and the operation temperature of gas sensor, has been studied in this work.

It is worthy of note that sol-gel deposition efficiency surpasses other chemical techniques, decreasing the waste of reactants; moreover, the set-up does not require an expensive vacuum system for synthesis. Hence, by the sol-gel technique, low cost manufacturing of ZnO thin films can be guaranteed. Additionally, ZnO-based sensors obtained by this technique present high sensitivity, low cost, fast response rate, and easy synthesis.

## 2. Experimental Procedure

### 2.1. Films Preparation

The ZnO:Ru and ZnO:Cr thin solid films were prepared by the sol-gel method based on a non-alkoxide route. The coating solution was prepared from zinc acetate dehydrated (Zn(C_2_H_3_O_2_)_2_·2H_2_O, Alfa Aesar, 98%) dissolved in a mixture of 2-methoxyethanol (CH_3_OCH_2_CH_2_OH, Sigma-Aldrich, 98%) and monoethanolamine (MEA, (CH_2_CH_2_OH)NH_2_, Sigma-Aldrich, 98%) at a molar concentration of 0.6 M. The solution was stirred at room temperature during one hour until a homogeneous and transparent solution was obtained. Ruthenium (III) trichloride (RuCl_3_, Alfa, 98%) and chromium (III) acetylacetonate (C_15_H_21_CrO_6_, Alfa, 98%) dissolved in deionized water (H_2_O) were used as doping sources.

The ZnO:Ru and ZnO:Cr thin films were deposited on clean soda-lime glass substrates (2.5 cm × 2.5 cm) by a repeated dip-coating process, at room temperature. The following cleaning procedure was used: a five minutes washing in trichloroethylene (C_2_HCl_3_, Baker, 98%) to degrease the substrate, followed by five minutes in acetone (CH_3_COCH_3_, Baker, 98%), then five minutes in methyl alcohol (CH_3_OH, Baker, 98%), and finally, a drying under a nitrogen flow (N_2_, Praxair, 99%). All washing steps were carried out in an ultrasonic water bath.

In order to remove the residual solvents after every immersion, an annealing process, in air at 200 °C for 10 min, was carried out. Additionally, in order to diffuse the Cr or Ru into the films, an extra annealing process was performed at 450 °C in air for one 1 h. The films thickness was controlled by the immersions number; in this work, 6 immersions for all ZnO films, and one, three, and five immersions for the doping process, were used. Undoped ZnO films were deposited for comparison with the doped ZnO films, in order to analyze the effect of the doping elements (Cr and Ru) on the sensing properties.

### 2.2. Films Characterization

The structure characterization of all deposited films was performed from X-ray diffraction, XRD, by using a Siemens-Kristalloflex diffractometer, with a Cu-K_α1_ (λ = 0.15405 nm) radiation, and 2θ angles ranging from 20 to 80, with 0.05 steps.The thicknesses of the films were measured by using a profilometer KLA- Tencor P-15 (with a resolution of 0.15 nm) on a step chemically manufactured. The surface morphology of the films was observed by scanning electron microscope (SEM) by using a Carl Zeiss Auriga 39-16 equipment. Secondary ion mass spectrometry (SIMS) measurements were carried out by a CAMECA IMS-6F Ion Microprobe, equipped with a cesium ion gun and duoplasmatron ion sources.

### 2.3. Sensing Properties

**Figure 1.**
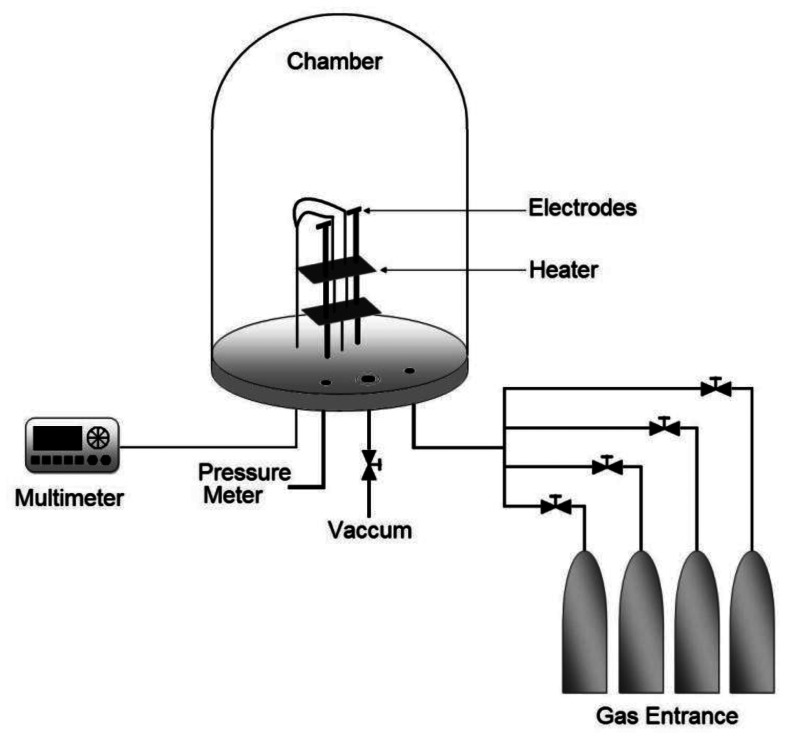
Schematic diagram of the system used to measure electrical properties in controlled atmospheres and temperatures.

Propane sensor characterization was carried out by placing the sensors in a sample-holder placed into a measurement chamber with a vacuum capacity of 10^−3^ Torr. The measurement chamber simultaneously allows the introduction of different gases in a controlled way. The diagram of the characterization system is shown in Figure 1. The ambient gas under consideration was zero-grade air (composition: O_2_ 19.5%–23.5%, H_2_O < 3 ppm, CO_2_ < 3 ppm, Total Hydrocarbon Content (THC) < 1 ppm, Praxair) and the gas being detected was propane (C_3_H_8_, Praxair).

Sensor characterization was performed by measuring the change in the electrical conductance (*ΔG*) of the film as a result of its interaction with the C_3_H_8_ being detected. This change in the electrical conductance was measured by a Keithley 2001 digital multimeter as a function of two main parameters: (a) temperature operation, under a given ambient gas concentration, which allowed the determination of the optimal operating temperature sensor, and (b) variations on C_3_H_8_ concentration at a constant operation temperature, which allowed an adequate determination of the sensor sensitivity (*S*). Last parameter can be adequately expressed as a function of the relative difference of the electrical conductances, according to the following equation:

(1) $$S=\frac{G_{G}-G_{O}}{G_{O}}$$

where G_G_ and G_O_ are the electrical conductance (1/electrical resistance) of the ZnO films measured in propane and air, respectively. In both ZnO:Cr and ZnO:Ru films, changes in the electrical conductance of the sample as a function of the exposition time to the C_3_H_8_ were measured, and after stabilization a constant value or a saturation value was reached. It is noteworthy that the response time of a sensor is defined as the required time to reach 90% of its saturation value (in the present case, for the *ΔG* parameter). Once this process was achieved, the detected gas was removed from the ambient atmosphere in a sudden way to determine the reversibility of the detection process. If the detection process shows reversibility, then the electrical conductance of the sample will exhibit the same value it had before propane exposition.

## 3. Results and Discussion

The structural, morphological and sensing characteristics of the films are presented in the following sections. The thicknesses measured for the one, three, and five immersions films, were around 80, 120 and, 180 nm, for both ZnO:Cr and ZnO:Ru thin films. The surface profile or the rms roughness of the films was measured, and values between 10–20 nm were estimated with an accuracy of 10%.

### 3.1. Structural Properties

**Figure 2.**
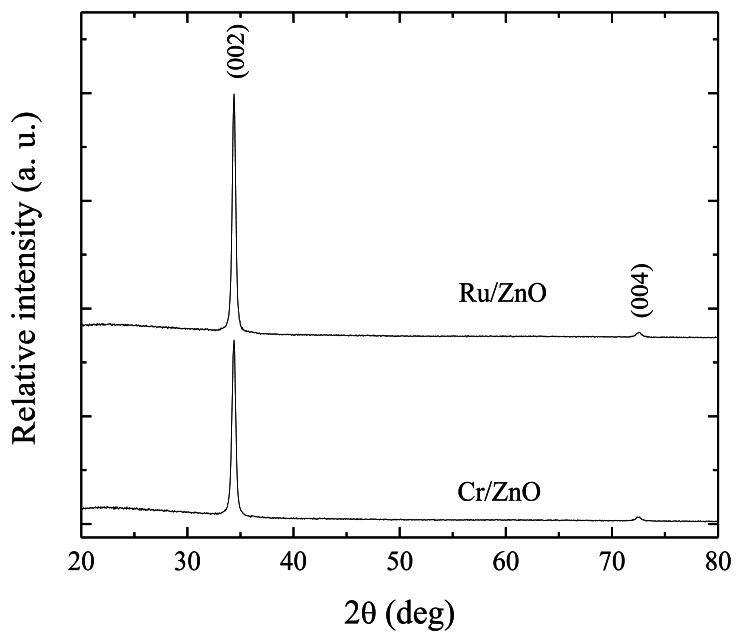
X-ray diffraction patterns of ZnO:Cr and ZnO:Ru thin films.

Figure 2 shows the X-ray diffraction patterns for the three immersions ZnO:Ru and ZnO:Cr samples. The two peaks presented can be perfectly indexed to the hexagonal wurtzite structure. The presence of a prominent peak, corresponding to (002) planes shows that the films are highly oriented along the c-axis. The (004) peak (2θ = 72.56) with a very low intensity, as compared with the (002) peak, is present in both spectra. The ZnO lattice constants estimated (a = 3.2499 Å and c = 5.2065 Å), for both thin films, are consistent with the bulk ZnO (JCPDS card No. 36-1451) [26].

Additionally, for the two samples no diffraction peaks from other elements or compounds were presented in the patterns. The average crystallite sizes were estimated from Debye Scherrer formula [27]:

(2) $$D=\frac{0.9\lambda }{B\cos\theta }$$

where *D* is the crystallite size in nanometers, *λ* is the wavelength value of the Cu-K_α1_ line (*λ* = 0.154056 nm), *θ* is the Bragg diffraction angle, and *B* is the FWHM of the diffraction peak measured in radians. The values were around 20 and 16 nm, for ZnO:Ru, And ZnO:Cr thin films with an accuracy of 10%, correspondingly.

**Figure 3.**
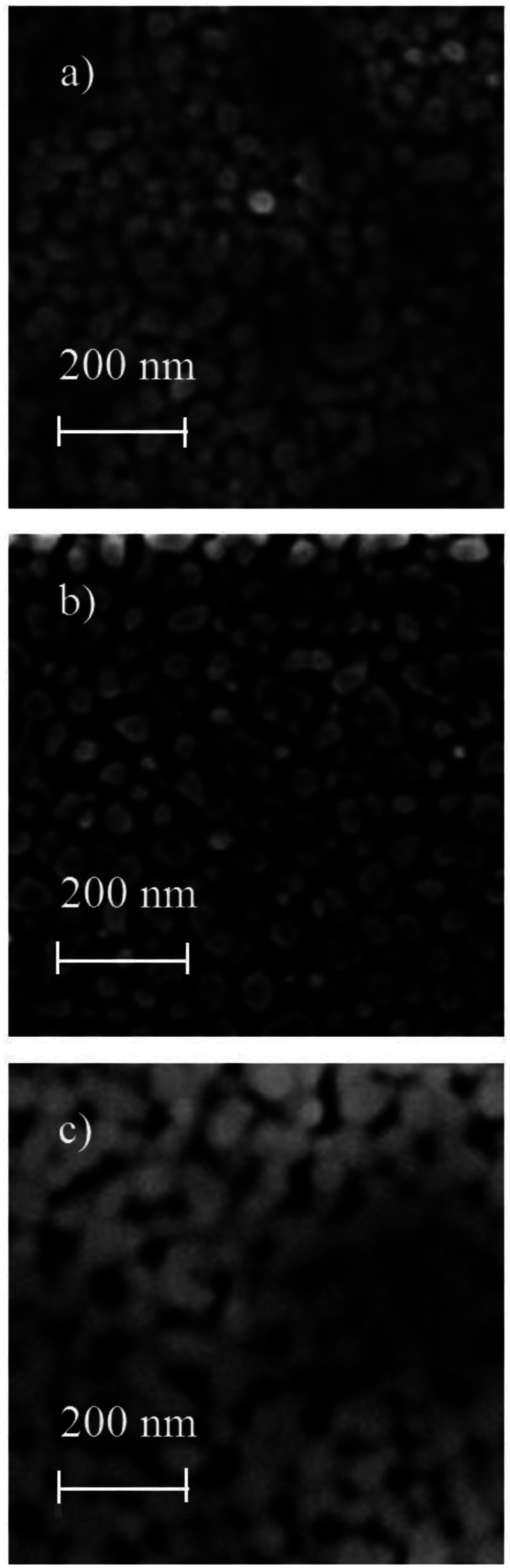
SEM images of ZnO:Cr thin films with different immersions number: (**a**) one, (**b**) three, and (**c**) five immersions.

**Figure 4.**
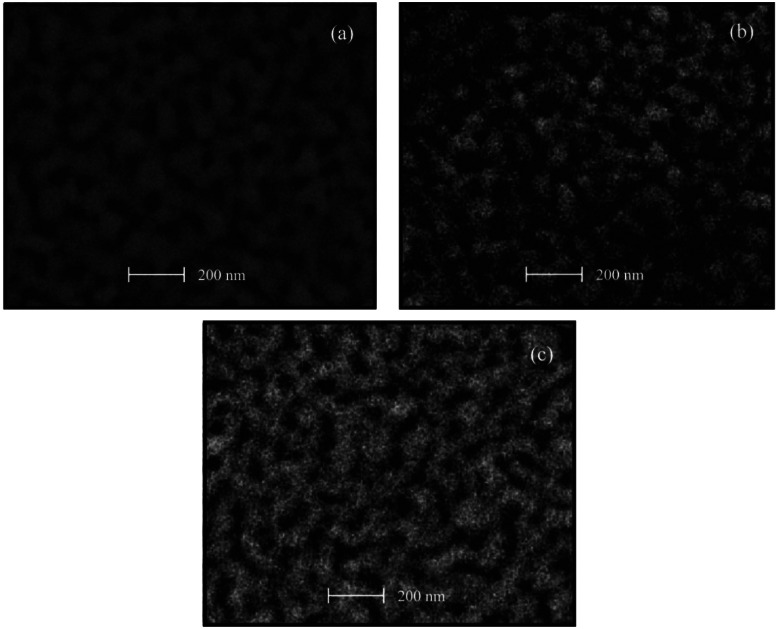
SEM images of ZnO:Ru films with different immersions number: (**a**) one, (**b**) three, and (**c**) five immersions.

Figures 3 and 4 show the SEM images of ZnO:Cr and ZnO:Ru films, respectively. As can be seen, in general, ZnO:Cr and ZnO:Ru thin films show a granular and porous surface morphology, with grain sizes varying between 30 and 50 nm in diameter, in both cases. Figure 3(a–c), correspond to ZnO:Cr thin films with one, three, and five immersions in the Cr solution, respectively. Figure 3(a,b) images shows a surface covered by rounded grains around 50 nm in diameter, with uniform distribution of small holes. Comparing image Figure 3(c) with images Figure 3(a,b), image Figure 3(c) presents a surface less compact with bigger grains (all around 55 nm), then, the porosity is more evident. The surface seems to be covered by rounded grains that are connected among them, forming linked chains from agglomerates of grains.

The SEM images shown in Figure 4(a–c) present surfaces relatively rough, then, in these films a higher surface area, as compared to the SEM images of the ZnO:Cr films, is obtained. Additionally, all samples seem to be homogenous with similar compactness. Figure 4(a) exhibits a closely packed spherical grain surface; however the formation of a nodular structure from these spherical grains can be evidenced as well. The average grain size in this one immersion ZnO:Ru film is around 40 nm. With respect to the surface morphology of the three immersions ZnO:Ru film shown in Figure 4(b) the image lacks of definition, but it seems to be covered with more irregular shape and grain size. The average grain size estimated is around 50 nm. Finally, Figure 4(c) shows the SEM micrograph of the five immersions ZnO:Ru film, where an uniform distribution of holes and a higher nodular configuration, as compared with Figure 4(b), was evidenced. The average grain size is in the order of 40 nm.

### 3.2. Secondary Ion Mass Spectrometry Measurements

**Figure 5.**
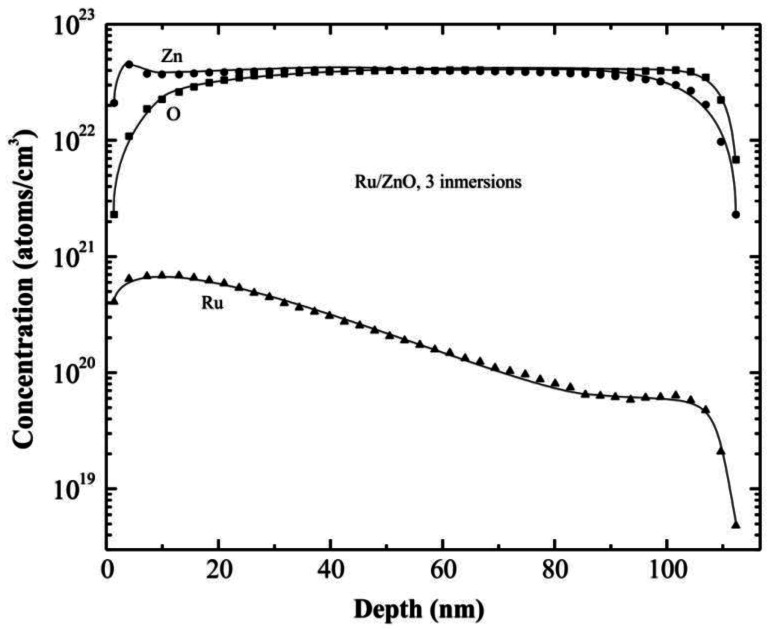
SIMS depth profiles for the three immersions ZnO:Ru film.

In order to prove the incorporation of the impurities into the films, a depth profile of Ru on a ZnO:Ru thin film, using Secondary Ion Mass Spectrometry (SIMS), has been developed. The profile shown in Figure 5 corresponds to the three immersions in Ru film, with a thickness around 120 nm. From the results obtained, it is evident that Ru was effectively incorporated to the ZnO films, however the Ru concentration decreases as the depth increases. Regarding the Ru particles diffusion into the films, which is assisted by the thermal process, it may be through both intergranular zones and porous in the ZnO film previously deposited. Then, at a higher depth less Ru can be encountered.

From the SIMS profiles it is possible estimate the thickness of the films; in this case it is around 100 nm, whereas the thickness measured directly from profilometry was around 120 nm.

### 3.3. Sensing Properties

**Figure 6.**
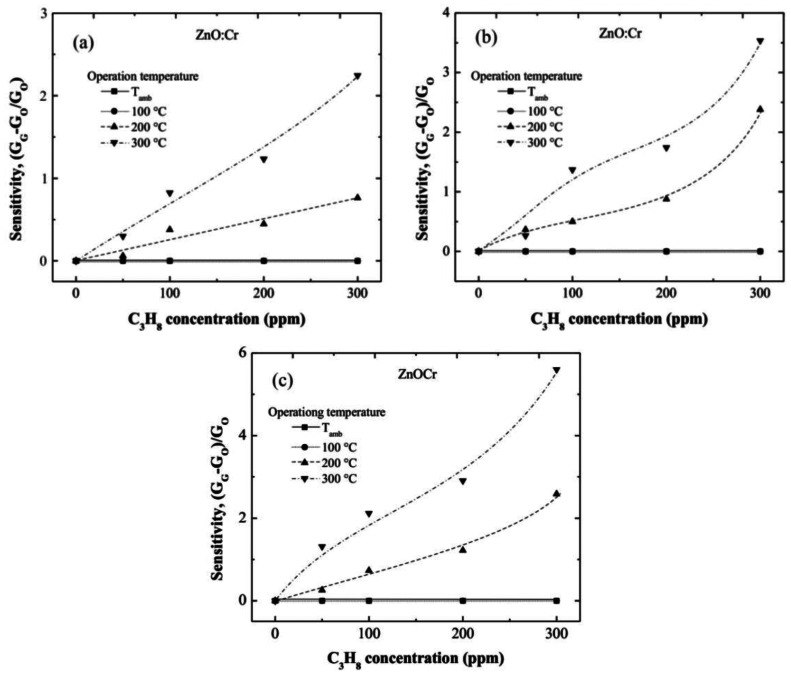
Sensitivity as a function of C_3_H_8_ concentration for ZnO:Cr films with different Cr content, (**a**) one, (**b**) three, and (**c**) five immersions; measured at room temperature, 100, 200 and 300 °C.

**Figure 7.**
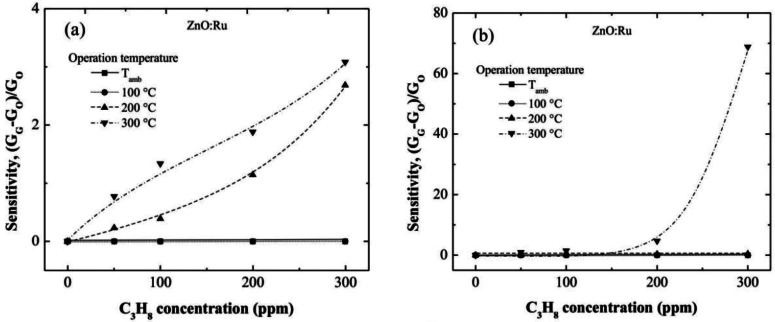

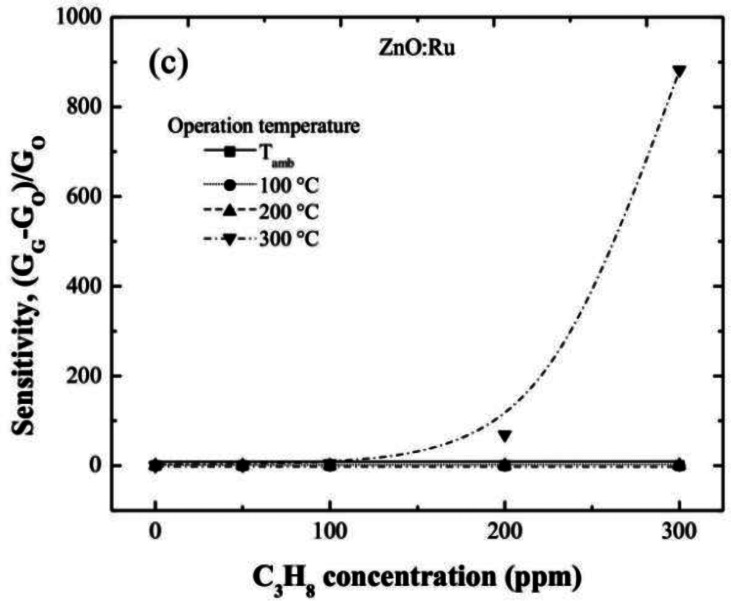
Sensitivity as a function of C_3_H_8_ concentration for ZnO:Ru films with different Ru content, (**a**) one, (**b**) three, and (**c**) five immersions; measured at room temperature, 100, 200 and 300 °C.

In this section is shown the enhancement of the sensing properties of the films with the incorporation of Ru and Cr. The sensing characteristics of the ZnO:Cr and ZnO:Ru films as a function of the operating temperature and C_3_H_8_ concentration are shown in Figures 6 and 7, respectively.

### 3.4. Characterization of ZnO:Cr Films

In general, all films are clearly sensible to both operation temperature and C_3_H_8_ concentration, nevertheless at values lower than 100 °C no resistance changes were registered. This result is due to the thermal energy is not enough for producing the desorption reactions that lead to the reduction of the surface. At higher temperatures, 200 °C up, important resistance changes were observed. This result is associated to the desorption of surface oxygen on the ZnO:Cr film, which takes place at higher operation temperatures.

Figure 6(a–c) correspond to one, three, and five immersions in the Cr solution, respectively. From the tendencies observed in the three graphs, it can be observed that sensitivity increases with the immersions number in the Cr solution. This result proves the catalytic effect of the Cr; and considering that the Cr content increases with the number of immersions in the Cr solution, then, one may conclude that the more Cr content the more the film sensitivity.

The maximum sensitivities registered at 300 ppm of C_3_H_8_, measured at 300 °C, were around 2.25, 3.6, and 5.8, for the one, three, and five immersions ZnO:Cr films, respectively. The role of the dopant, Cr in this case, produces an injection of electrons in the surface region due to the oxidation of the C_3_H_8_, as a consequence of the different between the work functions of the dopant [28] (Cr: 3.95–4.21 eV) [29] and the ZnO (4.5 eV) [30].

Until a content of 300 ppm of C_3_H_8_, the ZnO:Cr films sensitivity does not present a saturation effect, that is indicative of remaining surface oxygen for additional desorption, and consequently more desorption reactions can be carried out at higher temperatures.

The maximum sensitivity, registered in the three immersions ZnO:Cr film, can be attributed to the most porous film obtained at these deposition conditions, as can be confirmed from the SEM image 4(c).

### 3.5. Characterization of ZnO:Ru Films

Figure 7(a–c) show the sensitivity values estimated from electrical conductance measured in ZnO:Ru films doped from different immersions number in the Cr solutions. The sensitivity values estimated from 100 and 200 °C measurements were very low compared with those obtained at 300 °C. Additionally, the sensitivity magnitudes obtained at an operating temperature of 300 °C were outstandingly higher than those obtained in ZnO:Cr films at the same measurement conditions. This result confirms that Ru acts as a better catalyst than Cr in ZnO films deposited by the sol-gel technique.

The maximum sensitivities obtained for the one, three, and five immersions ZnO:Ru samples, were 3, 69, and 890, respectively, measured all at 300 °C.

### 3.6. Characterization of ZnO Films

**Figure 8.**
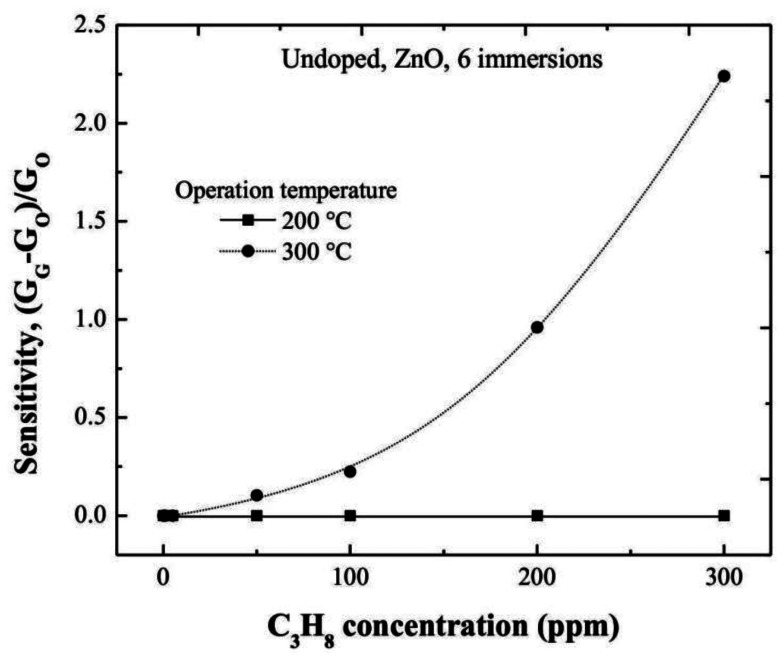
Sensitivity as a function of C_3_H_8_ concentration for the undoped ZnO film measured 200 and 300 °C.

Undoped ZnO thin films, also deposited from six dipping or immersions in the Zn starting solution by the sol-gel technique, were used as reference. In order to get the sensing characteristics of undoped ZnO films, and to evidence the catalytic effect of the Cr and Ru, films were characterized in the same way at two different operating temperatures, namely, 200 and 300 °C. The trend of the sensitivity values with the C_3_H_8_ concentration is shown in Figure 8. The maximum sensitivity measured at 300 °C, was around 2.3 for the maximum C_3_H_8_ concentration used in this work, 300 ppm.

It is noteworthy that the response time of a sensor is defined as the required time to reach 90% of its saturation value (in the present case, for the *ΔG* parameter), with a response time in the order of 60 s with an accuracy of 10%. Therefore, it is evident the positive effect of the Cr and Ru on the sensing properties of the ZnO thin films.

## 4. Conclusions

We have successfully deposited ZnO, ZnO:Cr, and ZnO:Ru thin solid films by the dip-coating sol-gel technique on soda-lime glass substrates. Deposition of all samples were reached by six immersions in a 0.6 M starting solution, prepared from zinc acetate, and then ZnO films were doped by an additional process consisting in one, three or five immersions of the ZnO films into a solution containing Cr or Ru. All films showed both excellent adherence and stability. X-ray diffraction studies confirm the existence of a polycrystalline hexagonal wurtzite structure, with a (002) preferred orientation in all the deposited films, in spite of its low thickness. SEM images show rough and porous surfaces, with small grain size, and almost irrespective of the thickness magnitude. SIMS analysis confirms the Ru incorporation and shows the distribution along the ZnO film.

From the present investigation we have shown that Ru is a better candidate than Cr to be used as catalyst in ZnO films for detecting C_3_H_8_ gas. Additionally, it was shown that at higher catalyst content more sensitivity was registered. In this respect, the ZnO film with five immersions of Ru exhibited the highest sensitivity, around 890 for 300 ppm of C_3_H_8_, measured at a temperature of 300 °C. The results presented in this work suggest that the sensitivity of the undoped ZnO thin solid films were significantly improved by the Cr and Ru doping. The ZnO:Ru films were all polycrystalline, with a (002) preferential growth and with a porous surface, that enhances the gas sensing detection. The film thickness plays a key role in the gas sensing characteristics of ZnO:Ru, as an optimum response was found in films with the highest film thickness.
